# Golgi apparatus analyzed by cryo-electron microscopy

**DOI:** 10.1007/s00418-013-1136-3

**Published:** 2013-08-18

**Authors:** Hong-Mei Han, Cedric Bouchet-Marquis, Jan Huebinger, Markus Grabenbauer

**Affiliations:** 1 Department of Systemic Cell Biology, Max-Planck-Institute of Molecular Physiology, Otto-Hahn-Str. 11, 44227 Dortmund, Germany; 2 Department of Molecular Cellular and Developmental Biology, University of Colorado, Boulder, CO USA; 3 FEI Company, 5350 NE Dawson Creek Drive, Hillsboro, OR 97124 USA; 4 Institute of Anatomy and Cell Biology, Heidelberg University, INF 307, 69120 Heidelberg, Germany

**Keywords:** Golgi apparatus, High-pressure freezing (HPF), Self-pressurized rapid freezing (SPRF), Cryo-electron microscopy of vitreous sections (CEMOVIS), Freeze substitution

## Abstract

In 1898, the Golgi apparatus was discovered by light microscopy, and since the 1950s, the ultrastructure composition is known by electron microscopic investigation. The complex three-dimensional morphology fascinated researchers and was sometimes even the driving force to develop novel visualization techniques. However, the highly dynamic membrane systems of Golgi apparatus are delicate and prone to fixation artifacts. Therefore, the understanding of Golgi morphology and its function has been improved significantly with the development of better preparation methods. Nowadays, cryo-fixation is the method of choice to arrest instantly all dynamic and physiological processes inside cells, tissues, and small organisms. Embedded in amorphous ice, such samples can be further processed by freeze substitution or directly analyzed in their fully hydrated state by cryo-electron microscopy and tomography. Even though the overall morphology of vitrified Golgi stacks is comparable to well-prepared and resin-embedded samples, previously unknown structural details can be observed solely based on their native density. At this point, any further improvement of sample preparation would gain novel insights, perhaps not in terms of general morphology, but on fine structural details of this dynamic organelle.

## Introduction

The era of ‘Golgi controversy’ between the light microscopic description of the internal apparatus by Golgi (1898) and the acceptance as a *bona fide* organelle with ‘lamellar structure’ in the 1950s is irritating from today’s view (reviewed in: Farquhar and Palade 1981, 1998). The scientific community was divided into ‘believers’ and ‘non-believers,’ as there was no method for a direct observation of the organelle. At light microscopic level, the Golgi apparatus has been characterized by metallic impregnation methods—silver or osmium tetroxide (OsO_4_)—known to be prone to artifacts. Interestingly, our actual concept of Golgi architecture is also based on metallic impregnation methods, but at the electron microscopic level, as reviewed recently by Klumperman (2011). Two classical models of protein transport through the secretory pathway have dominated the Golgi field for more than five decades: the cisternal maturation/progression model (Grasse 1957) and the vesicular transport model (Jamieson and Palade 1967). Despite successful Golgi research in a great variety of scientific branches and techniques, this fundamental question remains unanswered. The membrane stacks, vesicles, COP, and clathrin coats are visualized in routine electron microscopy by the deposition of heavy metals like osmium, lead, and uranium. During such experiments, the membrane lipids, the ions, and the water content of the sample are removed completely during dehydration procedures and will not even enter the microscope. Therefore, we have to ‘believe’ that the observed osmicated structures resemble the organelle architecture in living cells.

The electron microscopy preparation and analysis methods developed further from so-called routine electron microscopy of resin (plastic) sections, as introduced in the early 1950s (Sjöstrand 1951), to cryo-based techniques employed far below room temperature. In the 1980s, the direct visualization of frozen-hydrated biological material by electron microscopy was established (McDowall et al. 1983; Dubochet et al. 1988), with the very origins dating back to early 1970s (Christensen 1971). Since the last three decades, cryo-electron microscopy of vitrified sections (CEMOVIS) gradually developed to a stage, where it can be applied to various biological specimens (Al-Amoudi et al. 2004; Dubochet et al. 2007), and provided unprecedented views of different structures in their native cellular environment, such as microtubules (Bouchet-Marquis et al. 2007), desmosomes (Al-Amoudi et al. 2007), mitochondria (Hsieh et al. 2006), and neuronal synapses (Zuber et al. 2005). Studies on Golgi apparatus are still rare (Bouchet-Marquis et al. 2008), and very often published images of vitrified Golgi stacks are a side result of studies on special cell types or tissues (Henderson et al. 2007; Gruska et al. 2008). Actually, vitrified sectioning should not be seen as a competition to the more established plastic-section electron microscopy, but constitutes an excellent complement, filling in high levels of structural details in the overview of cellular architecture (Bouchet-Marquis and Hoenger 2011). In this review, we will focus on the influences of fixation on the architecture of Golgi apparatus, how it is seen by cryo-electron microscopy, as well as shedding light on what we have learned from these results, how structural data from single-particle reconstructions and sub-tomogram averaging could fit in, and what to expect from future technical developments in cryo-microscopy like non-slicing procedures.

## Golgi morphology after chemical fixation

Before the introduction of aldehyde fixation for electron microscopy in the early 1960s (Holt and Hicks 1961; Sabatini et al. 1963), samples were generally fixed by metal salts like osmium tetroxide (OsO_4_), whereas the permanganate fixation showed slight advantages on Golgi morphology (Mollenhauer and Zebrun 1960). Using OsO_4_ fixation, the general architecture of Golgi apparatus—sometimes named ‘dictyosome’—consisting of stacked membranes and surrounding vesicles was described at impressive clarity in sea urchin eggs (Afzelius 1956) and exocrine cells of murine pancreas (Sjöstrand and Hanzon 1954b), with their important fixation details published elsewhere (Sjöstrand and Hanzon 1954a, c). After the aldehyde fixation being established in the 1960s, electron microscopic research developed toward cytochemical labeling, which allowed to understand the functional morphology and *cis*–*trans* polarity by differential distribution of glycosylation enzymes along the Golgi stacks (Farquhar and Palade 1981). Therefore, the main focus was on preserving enzyme activities after fixation (Novikoff et al. 1961) and also on retaining antigenicity [(Zhdanov et al. 1965; Sternberger and Donati 1966) reviewed in (Roth 1996)], taking slight compromises on the morphological appearance of the whole organelle into account.

Highlighting all aldehyde-fixation-based cytochemical methods like immunolocalization of antigens (Roth 1996; Rabouille and Klumperman 2005), correlative light and electron microscopy, and even three-dimensional protein localization (Grabenbauer et al. 2005; Zeuschner et al. 2006) would be beyond the scope of this article and was recently reviewed (Klumperman 2011). However, it should be noted that compromises on fixation procedures could lead to artifacts like very small membrane connections between adjacent cisternae. The misinterpretation of such results might induce wrong concepts on the functional morphology of Golgi apparatus. Nevertheless, our knowledge on the general Golgi morphology as described in current textbooks is based on impressive studies of the aldehyde-fixed organelle investigated in three dimensions (Ladinsky et al. 1994; Soto et al. 1994).

## Cryo-fixation: plunge freezing

A major improvement on the fixation of biological specimen occurred with the introduction of cryo-fixation. Compared to chemical aldehyde reactions, which crosslink biological material in a timescale of seconds to minutes, the velocity of cryo-fixation is far superior, since all biochemical, physiological, and dynamic processes are arrested during 10–20 ms in their actual state by a massive temperature drop. The viscosity increases dramatically and the sample transforms into a ‘glass’—meaning that it is completely embedded in vitreous or sometimes called amorphous ice. The vitreous specimens remain fully hydrated and are still liquid by physical definition, but share properties of solid matter as they are in fact in a very high viscosity state. However, the process of vitrification is not completely understood yet. Constantly kept below the devitrification temperature of −140 °C (for pure water), the ‘glassy’ sample will not flow in the timescale of a realistic experiment, even viewed at electron microscopic magnifications (Dubochet et al. 2007; Dubochet 2007). To ensure the transition of the sample to a ‘glassy state’ and avoid any segregation of molecules by ice crystal growth, a cooling velocity up to 100,000 °C/s is intended. Thin samples such as purified macromolecules deposited on an electron microscopy grid, very small cells like bacteria or flat parts of eukaryotic cell periphery can be completely vitrified by simply plunge freezing them into an adequate cryogen (Dobro et al. 2010).

The main advantage of studies performed on vitrified specimens is the preservation of their inherent native densities, revealing the natural arrangements of biological structures. This means, we ‘see’ and image directly the membranes, ribosomes, fibers, protein complexes, and larger molecules by a direct interaction between those biological structures and the electron beam, not by a secondary detection of an artificial heavy metal impregnation (Sartori Blanc et al. 1998; Dubochet et al. 2007). Furthermore, aggregation of biological structures—a phenomenon usually attributed to the dehydration process during resin embedding—is dramatically reduced.

Among the various approaches developed for cryo-fixation, plunge freezing is one of the earliest (Taylor and Glaeser 1976) and was already used for the first electron microscopic characterization of the vitrification of pure water (Dubochet and McDowall 1981; reviewed in: Dubochet 2012). For samples like larger cells or tissues, where the Golgi field is located in the perinuclear region, this technique is not suitable, as the freezing speed decreases rapidly from sample surface deeper into the bulk, inducing ice crystal growth (Studer et al. 2008). But for small prokaryotic cells, plunge freezing is a surpassing fixation procedure leading to well-vitrified samples. Hence, the general view of bacteria, formerly seen as a ‘bag of enzymes’ was revolutionized by cryo-electron tomography of plunge-frozen samples, and today’s understanding of cellular substructures like complex bacterial cytoskeleton and the architecture of various large macromolecular complexes emerged (Chen et al. 2010; Pilhofer et al. 2010; Gan and Jensen 2012).

**Fig. 1:**
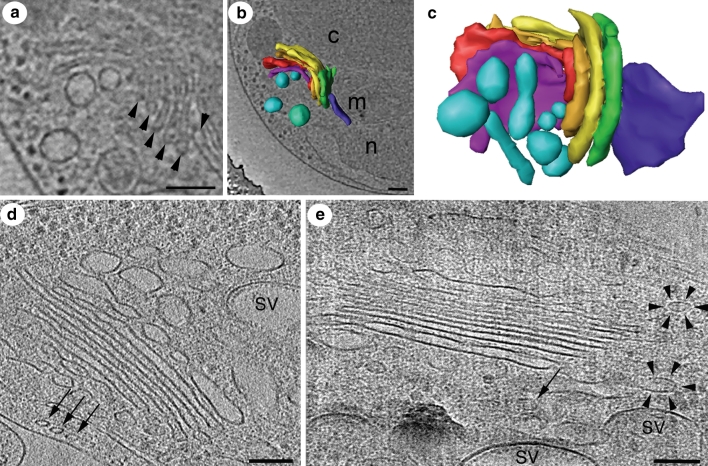
Cryo-tomograms of Golgi apparatus in unicellular organisms. **a**–**c** Golgi apparatus of green alga *Ostreococcus tauri*. **a** A slice through the tomogram shows five Golgi cisternae, marked by arrowheads. **b** The 3D segmentation of Golgi apparatus is shown in situ within its cellular context. The ‘core’ cisternae are colored in *deep purple*, *red*, *gold*, *yellow*, and *green* (*cis* to *trans*), surrounding vesicles in *light* and *dark blue*. **c** Isolated 3D segmentation is shown from a perpendicular view with the same color code as in **b**. The blue-green vesicle in **b** was removed to create an unobstructed view of the Golgi stack. *c* chloroplast, *m* mitochondrium, *n* nucleus. **d**–**e** Golgi apparatus of unicellular parasite *Trypanosoma brucei* (Kinetoplastida). **d** The Golgi stack consists of 7–8 cisternae. Note the subpellicular microtubule arrangement below the plasma membrane (*arrows*). Secretory vesicles (SV) mark the *trans* side of Golgi stack. **e** In some tomographic slices, the COP coat is visible in a ‘spiky appearance’ (*arrowheads*). **a**–**c** are adapted from (Henderson et al. 2007). *Scale bars* 100 nm

One of the smallest known eukaryotic cells is *Ostreococcis tauri*, a unicellular green alga containing a single mitochondrium, one chloroplast, and one Golgi apparatus (Courties et al. 1994). It is the only eukaryotic cell so far, which was effectively imaged in its entity by cryo-electron microscopy and tomography (Henderson et al. 2007) (see Fig. 1a–c). Being able to plunge freeze and image directly the cells by electron microscopy, many steps of sample preparation are avoided, like postfixation, dehydration, embedding, sectioning, and staining, getting around all their potentially related artifacts. In high-quality cryo-tomograms, single Golgi stacks per cell have been identified, consisting of five cisternae without any luminal contacts and a low amount of peri-Golgi vesicles (Henderson et al. 2007).

## High-pressure freeze fixation

**Fig. 2:**
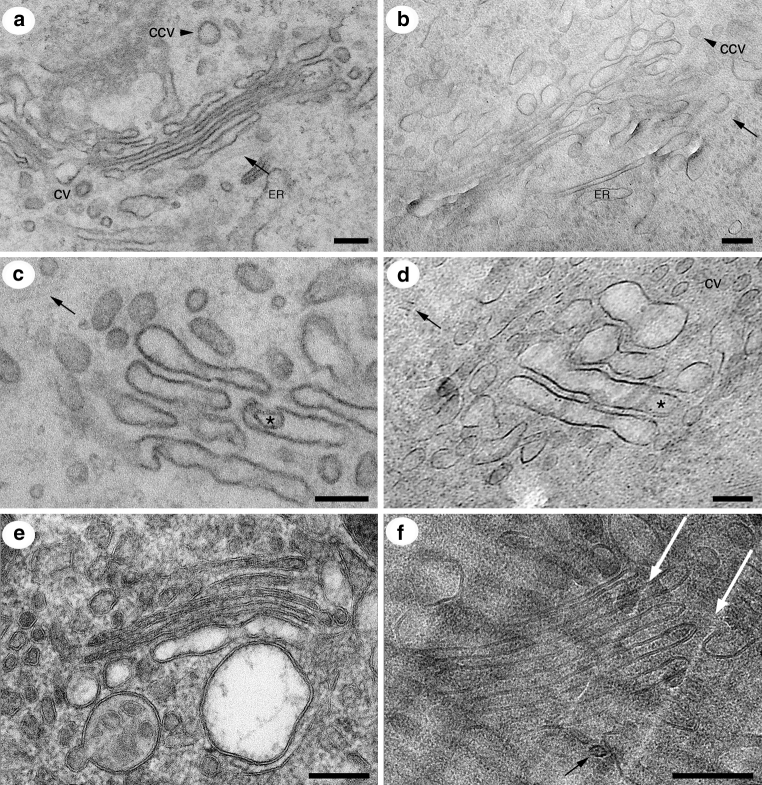
Golgi apparatus of cultured mammalian cells in vitreous ice compared to chemical-fixed, dehydrated, and plastic-embedded specimens. **a** Overview of glutaraldehyde-fixed, osmicated, and lead-stained Golgi apparatus. The coats of clathrin-coated vesicles (CCV) and COP-coated vesicles (CV) exhibit high contrast. Without uranyl staining, microtubules are hardly visible (*arrows*). Endoplasmic reticulum (ER) is recognized by attached ribosomes. **b** Golgi apparatus in vitreous sections exhibits less contrast. A clathrin-coated vesicle (CCV) is located at the *trans* side. **c** Higher magnification of Golgi stack in conventional preparation. Membraneous invaginations into Golgi cisternae (*asterisk*)—often regarded as fixation artifact—appear similar to vitreous preparations (see *asterisk* in **d**). **d** Coated peri-Golgi vesicles (CV) next to Golgi cisternae appear ellipsoid due to compression during vitreous sectioning. Note the part of a microtubule (*arrow*). **e** Golgi apparatus after high-pressure freeze fixation and freeze substitution. Membranes appear ‘smoother’ and the lipid bilayer is clearly visible. **f** In vitrified sections, the membrane contrast is directly based on the presence of organic biological material like lipids and proteins, not on heavy metal impregnation. Note that protofilaments of a microtubule are well resolved (*arrow*), and the section is slightly compressed in the direction of cutting (see knife marks in direction of *white arrows*). The stacks in **a**–**d** are oriented with *cis* side at the bottom and *trans* on top of the image, while **e**, **f** display the opposite orientation. **a**–**d** are reproduced from (Bouchet-Marquis et al. 2008) with permission from John Wiley & Sons Inc. *Scale bars* 100 nm

Reaching a proper vitrification of samples in the size of most eukaryotic cells and tissues requires the use of high-pressure freezing (HPF)—as introduced by Riehle and Moor (Moor and Riehle 1968; Moor 1987)—and is nowadays performed through commercially available HPF machines. In cryo-technical terms, the most important effect of high pressure is a reduction in the cooling rate required to vitrify the sample. Compared to plunge freezing, the sample thickness, which can be properly vitrified, is extended from a few micrometers to 200–300 μm (Studer et al. 2008). Recently, self-pressurized rapid freezing (SPRF) was established as a novel and low-cost cryo-fixation method to freeze biological samples in copper tubes that are clamp-sealed on both sides. Instead of applying about 2,000 bar pressure and synchronous cooling in a HPF apparatus, the tubes were plunged directly into the cryogen. In parts of the tube, crystalline ice is formed and builds up pressure sufficient for the liquid–glass transition of the remaining specimen. This relatively simple procedure—as compared to the usage of HPF machines—provided at first good-quality results for freeze-substituted and resin-embedded specimens (Leunissen and Yi 2009). By cryo-electron microscopy and electron diffraction, it was further determined that adjusted freezing conditions in SPRF result in vitreous samples of comparably high-quality to HPF machines (Han et al. 2012). Both cryo-fixation methods—HPF and SPRF—arrest biological samples up to 100–300 μm thickness instantly in vitreous ice, which can be further processed through freeze substitution and subsequent resin embedding, or can be imaged directly by cryo-electron microscopy of vitreous sections (CEMOVIS) (see Fig. 2).

For all the facets of sample preparation by HPF followed by freeze substitution, we have to refer to available protocols (Buser and Walther 2008) and excellent reviews (McDonald 1999, 2007). However, it should be noted that the ‘Boulder Laboratory for 3DEM of cells’ set benchmark studies on visualizing Golgi apparatus complexity, based on freeze-substituted specimen analyzed by serial sectioning and dual-axis electron tomography (Ladinsky et al. 1999; Marsh et al. 2001).

Compared to conventional ‘dry’ and heavy metal-stained plastic sections, cryo-electron microscopy and tomography add a substantial part of complexity in preparation, imaging, and interpretation of the images obtained. First, the apparent contrast of the biological material embedded in amorphous ice is lower (see Fig. 2). Second, vitrified samples are beam-sensitive and the electron dose has to be limited. Therefore, the resulting cryo-electron tomograms are often characterized by a very low signal-to-noise ratio. However, due to the recent improvements in computer-controlled cryo-electron microscopes, CCD, and CMOS cameras as well as image processing software, a resolution of several (4–5) nanometers can be obtained when performing cryo-electron tomography on vitreous biological samples (Nickell et al. 2006; Bouchet-Marquis and Hoenger 2011; Diebolder et al. 2012).

## Golgi apparatus in vitreous sections

**Fig. 3:**
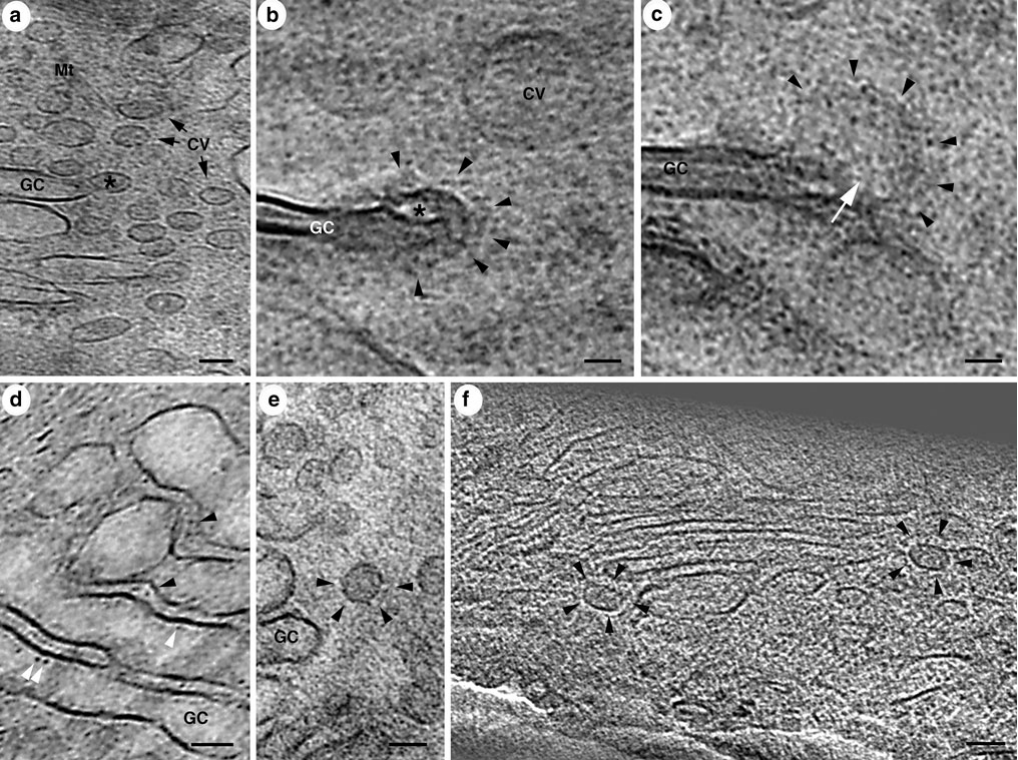
Structural details of vitreous Golgi apparatus in human cells (HeLa). **a** Some coats exhibit a ‘spiky’ substructure on coated peri-Golgi vesicles (CV) and coated buds (*asterisk*) emanating from Golgi cisternae (GC). Distance between spikes is 8–9 nm. **b** Higher magnification view of a budding profile with more homogeneous coat (*arrow heads*). **c** Budding profile on the rim of a Golgi cisterna (GC). The coat is hardly visible (*arrow heads*). The *white arrow* points to a structure probably involved in the budding process. **d** Golgi cisternae from Fig. 2d in higher magnification. Protein complexes in the cisternal lumen (*white arrow heads*) as well as in the cisternal cleft (*black arrow heads*) are visible by their biological contrast. **e** Clathrin-coated vesicle (see CCV in Fig. 2b) at higher magnification. The clathrin cage around the vesicle is marked with *black arrowheads*. **f** Tomographic slice of a 3D reconstruction containing Golgi apparatus at the periphery. The ‘spiky coats’ of two vesicles are clearly visible. **a**–**e** are reproduced from (Bouchet-Marquis et al. 2008) with permission from John Wiley & Sons Inc. *Scale bar* in **a** 50 nm; **b** 20 nm; **d**, **e** 50 nm

The application of CEMOVIS enables to resolve the details of Golgi apparatus formerly hidden in plastic-section electron microscopy (Bouchet-Marquis et al. 2008). The general morphology of the organelle with 4–5 separate cisternae surrounded by peri-Golgi vesicles shows—as expected—no structural differences between freeze-substituted versus vitreous material of the same cells (see Fig. 2). At low-to-medium electron microscopic magnification (1,000*x*–20,000*x*), well-contrasted plastic sections give even substantial advantages compared to vitreous sections. At higher magnification, known structures like vitrified clathrin coats give gentle contrast in their cellular environment, compared to purified samples in cryo-electron microscopy (Cheng et al. 2007). The COP coats of peri-Golgi vesicles appear as homogeneous or ‘spiky’ subtypes, whereas it remains unclear, if ‘spiky coats’ and ‘homogeneous coats’ represent distinct subtypes differing in genesis and protein composition, or if they are COP coats at different stages of coating/uncoating (Bouchet-Marquis et al. 2008) (see Fig. 3). However, their presence in systematically and evolutionary highly separated organisms such as humans and—for Golgi research very interesting—trypanosomes (Kinetoplastida) (He et al. 2004, 2005; Warren 2013) shows their ubiquity in eukaryotic cells (see Fig. 1e). At least, a differentiation into COPIa and COPIb vesicles by size and content density—as recently introduced for freeze-substituted plant and algal cells (Donohoe et al. 2007)—can be neglected for mammalian and trypanosomal Golgi vesicles, since interior densities and sizes of the ‘spiky’ and homogeneous coated vesicles are not distinguishable. Strong evidence for functionally different COPI vesicles comes from biochemical (Malsam et al. 2005) and recent immunoelectron microscopic data (Béthune et al. 2006; Langer et al. 2007). However, it remains unclear, how slight composition differences would imply such structural diversification as observed by cryo-electron microscopy.

A new and unexpected finding at vitreous Golgi apparatus are protein complexes up to 6 nm in size and attached to cisternal membranes, as there is nothing comparable described in resin-embedded samples observed by electron microscopy, irrespective of freeze or chemical fixation and dehydration procedures. Their electron microscopic contrast is mainly phase contrast due to the wave function of electrons and related to the atomic potential distribution within biological molecules. After metal salt impregnation and dehydration, this phase contrast is concealed and overlapped by the amplitude contrast of stained material, detected through the particle behavior of electrons, whereas their phase contrast falls beyond detection (Dubochet et al. 2007; Bouchet-Marquis et al. 2008). Some small pleomorphic complexes are attached to the luminal side of cisternal membranes, while others are localized between adjacent cisternae and could have stabilizing functions, but only a profound structural analysis and comparison to known Golgi-localized proteins will clarify their composition and function (see Fig. 3d).

By cryo-electron microscopy, Golgi saccules have been shown 30–60 min after induction of procollagen secretion. This verified that such saccules exist also in vitreous ice-embedded samples and are not a sign of luminal swellings caused by local osmotic effects during inappropriate fixation or dehydration (Bouchet-Marquis et al. 2008). Furthermore, during massive cargo transport, a luminal connection between cisternae was detected by cryo-electron tomography in vitrified Golgi apparatus. This was an interesting finding concerning the recent discussion about tubular continuities between different Golgi cisternae. Some of them have been found in nocodazole-treated cells undergoing a cargo wave of viral proteins, which was released after a low temperature-induced traffic block—of course a highly non-physiological experimental setup (Trucco et al. 2004). In contrast to the first description of such tubules occurring during glucose-stimulated insulin secretion, with connections bypassing interceding cisternae in freeze-substituted samples (Marsh et al. 2004), the cryo-electron microscopic data showed only one luminal continuity very central in a cisterna at a place of potential branching of the Golgi stack (Bouchet-Marquis et al. 2008). After revisiting the original cryo-tomograms, it became clear that much more and larger Golgi areas have to be analyzed to get conclusive evidence. Unfortunately, further and unambiguous luminal connections could not be traced after analyzing all cryo-tomograms of these experiments. In contrast, the cisternae seemed to be well separated from each other (unpublished results). However, the unique cisternal continuity detected in this cryo-tomogram was also very different from other luminal continuities between adjacent cisternae as described during synchronized viral cargo waves (Trucco et al. 2004), as these were located at the outermost rim of medial cisternae, but not in the center of the stack.

## Technical considerations on vitreous sections

Cryo-electron microscopy of vitreous sections is a highly challenging technique, and studies specifically directed to understand Golgi morphology are rare (Bouchet-Marquis et al. 2008). Mostly, Golgi imaging is presented as side result of studies on technical improvements of imaging, or sometimes these results are well hidden in supplementary material (Gruska et al. 2008). The technical considerations of CEMOVIS seem to be straight forward with high-pressure freeze fixation of the cells, cryo-sectioning, and imaging—theoretically all done in 1 day. In practice, there are some challenges associated with this attempt. To achieve proper vitrification of cultured mammalian cells, usually a low cell number is used, resulting in block surfaces for cutting, which contain 1–5, or sometimes not even a single cell. The next issues reducing the success rate to find analyzable Golgi areas are the inherent cutting artifacts of vitreous sections: knife marks and compression. The latter effect is responsible for chatters and/or crevasses—breaks into the section, which occur at the surface when cutting thicker than 70 nm (Al-Amoudi et al. 2005; Dubochet et al. 2007; Bouchet-Marquis et al. 2008; Han et al. 2008; Bouchet-Marquis and Hoenger 2011). Crevassing happens when compression exceeds the levels that amorphous ice can sustain. In those cases, linear deformation of the ice is not an option anymore and results in breaks perpendicular to the surface of the section, which is detrimental to the cryo-tomogram quality from such areas of the cell. Crevasses can generally occur everywhere and cause discontinuous patterns, but are most disturbing as severe breaks in the track of biological membranes, which might abolish the proper analysis of complex membrane systems like Golgi apparatus. To study small and abundant organelles like mitochondria, fragmented cell areas showing unperturbed and well-fixed morphology might be sufficient to record reasonable amounts of data. But to have a juxtanuclear Golgi apparatus recognizable as such and in the right orientation for useful imaging, even in plastic sections containing hundreds of cells, one has to observe dozens to get acceptable areas. Next, one has to analyze whether the chosen cell is in good physiological condition, which might be judged on the morphology of additional organelles like mitochondria. During this selection in cryo-samples, there is always the danger for a less-experienced microscopist to destroy the area of interest just by electron beam irradiation during observation, even using the low-dose mode. Moreover, considering cryo-electron tomography, sometimes the best regions of interest would not be available for data collection because of the presence of nearby grid bars, surface contaminants, or ice crystals. Finally, as cryo-sections are mounted on the grid without the aid of liquids providing surface tension, they never flatten perfectly on the grid and sometimes remain flittering in the microscope, even if aided by electrostatic charging procedures (Pierson et al. 2010). Compared to plunge-frozen material, this leads to non-optimal conditions during tilt series recording and subsequent 3D reconstruction. Summarizing, for cryo-electron microscopic analysis of Golgi apparatus, a much higher number of cells has to be sampled compared to ‘more abundant’ organelles or structures like mitochondria or microtubules.

It should be mentioned here that gentle treatment of cells prior to fixation is often neglected and this might have severe impact on sensitive membrane systems like Golgi apparatus. No matter which improvements and efforts are done on the technical side, each image or 3D reconstruction is a representation of the sample’s physiological condition during fixation. Therefore, this part of the experiment should also be taken seriously. The treatment of cells immediately before cryo-fixation is a very critical point, where artifacts could and will be already induced (McDonald 1999, 2007; McDonald et al. 2010). To reach a ‘close-to-native’ state, one tries to keep cells as physiological as possible, which is already questionable for most mammalian cells outside a body. Next, a proper cryo-fixation for cryo-electron microscopy of cultured cells or tissues is dependent on the usage of cryo-protectants and application of high pressure during the freezing process, which are per se highly non-physiological conditions. The high pressure is a physical necessity to vitrify mammalian cells. As the pressure pulse is applied just milliseconds before freezing, we might neglect this point. However, we cannot neglect the usage of cryo-protectants, as these substances are mandatory for proper vitrification of mammalian cells. To achieve a sample quality convenient for CEMOVIS, one can vary the types of cryo-protectants, their concentrations, and/or the incubation times therein. Even mixtures of cryo-protectants might display advantages above their single constituents. This will always result in slightly decreased water content of the cells, even if the cryo-protectants are applied only briefly. But if total cell volume is reduced up to 20 % (unpublished observations), it is difficult, if not impossible, to delineate whether the volume reduction spreads evenly onto the whole cell or is more pronounced in cytoplasm/nucleoplasm or in some subcompartments like the secretory pathway. The volume decrease in cytoplasm is accompanied by an increase in osmotically active substances therein, which might even lead to a compensatory swelling of organelles like whole Golgi apparatus or just certain subcompartments like subsets of cisternae or vesicles. This is speculative indeed, as our current knowledge is very limited about the ‘real-native’ architecture of Golgi apparatus, its osmotic regulation, and their dynamics. Further, the physiological ion contents of all Golgi subcompartments are as well unknown yet. To circumvent any cryo-protectant-induced cellular volume decrease immediately prior to fixation, more research is needed. New approaches in HPF for cryo-electron microscopy, which give more flexibility than routine HPF machines, might lead to improved and more physiological freezing conditions (Han et al. 2012), probably with the option to once omit cryo-protectants completely.

Regarding all drawbacks of CEMOVIS, it is still the only way to observe organelles or biological structures directly at the molecular level in their ‘close-to-native’ cellular environment (Bouchet-Marquis et al. 2008; Bouchet-Marquis and Hoenger 2011).

## Golgi apparatus in non-sliced cryo-samples

Apart from cryo-electron tomography of plunge-frozen samples, as mentioned above on the example of *O. tauri* (Henderson et al. 2007), there exists further cryo-microscopic methods preventing the need for sample sectioning by cryo-ultramicrotomy. Cryo-soft X-ray tomography acquires images of frozen-hydrated samples in an absorption contrast mode. In the wavelength of 2.3–4.4 nm, organic material absorbs strongly against water and allows for recording tilt series of specimen up to 15 μm in depth. The resolution in biological material is about 36–70 nm (equivalent to 18–35 nm ‘half-pitch resolution’) (Schneider et al. 2010; McDermott et al. 2012a, b). Golgi apparatus was visualized using cryo-soft X-ray tomography in adenocarcinoma cells (Schneider et al. 2010; Müller et al. 2012) and the unicellular green alga *Chlamydomonas reinhardtii* (Hummel et al. 2012). Cisternal shapes and possibly budding vesicles could be seen, but the size of these structures is close to the resolution limit (the width of a cisterna corresponds to 2–4 pixels), preventing the segmentation and 3D reconstruction of Golgi stacks.

In a similar resolution range as cryo-soft X-ray tomography is a technique recently introduced in biological research: focused ion beam (FIB) milling for serial block face imaging in the scanning electron microscope (SEM) (Heymann et al. 2006). Golgi structures have been visualized in conventionally embedded cultured mammalian cells (Murphy et al. 2011; Villinger et al. 2012). So far, cryo-FIB milling of frozen-hydrated specimen was applied without direct ultrastructure imaging as a preparatory step for thinning samples to a suitable size for cryo-transmission electron tomography, instead of cryo-sectioning (Marko et al. 2006; Hayles et al. 2010; Rigort et al. 2010, 2012; Wang et al. 2012). With the improved detection of secondary electrons and with better understanding of their contrast formation on frozen-hydrated samples (de Winter et al. 2013), membranes might be visualized and we can expect 3D reconstructions of large tissue volumes in the frozen-hydrated state (personal communications: T. Landin, FEI company and A. Schertel, Carl Zeiss Microscopy), omitting all dehydration artifacts by performing slice and view imaging of cryo-fixed samples directly in the FIB/SEM.

## Golgi-derived samples studied by single-particle cryo-electron microscopy

Single-particle cryo-electron microscopy is a technique in structural biology that is widely used to solve the three-dimensional structures of isolated macromolecular assemblies close to their biological conditions. The technique started from relatively simple ‘negatively stained’ material deposited on electron microscope grids, as described in a more than 1,300 times cited article (Brenner and Horne 1959). However, single-particle analysis quickly gained momentum after the introduction of plunge freezing to stabilize the sample in vitreous ice and subsequent cryo-electron microscopic examination in the fully hydrated state (Taylor and Glaeser 1976; Dubochet and McDowall 1981; reviewed in: Dobro et al. 2010). Recent improvements in cryo-electron microscopy and single-particle reconstruction methodologies (reviewed in: Frank 2009) led to the determination of biological molecules at near-atomic resolution (0.33–0.46 nm), most successful on viral capsid proteins (Hryc et al. 2011), and—by using single-electron counting detectors—on proteasomes (Li et al. 2013), and ribosomes (Bai et al. 2013). The Golgi apparatus as whole organelle is by far not accessible by these techniques, but important contributions from structural biology and especially from single-particle reconstruction techniques led to our current understanding of the architecture of coats on membrane-bound vesicles and cisternal buds (Faini et al. 2013).

Clathrin-coated vesicles are important structures of membrane trafficking in cells, in particular of cargo transport from *trans*-Golgi network to endosomes and from plasma membrane to endosomes during endocytosis, as well as in numerous specialized pathways with physiological relevance. Clathrin was the first membrane coat described and its characterization defined the prototype for membrane coat function that applied to other intracellular and Golgi-derived coats (Brodsky 2012). Our current view on clathrin coat architecture is based on outstanding reports by Fotin et al. (2004, 2006). For further reading, which would be beyond the scope of this review, we have to recommend excellent review articles (Cheng et al. 2007; Kirchhausen 2009; Brodsky 2012).

The intracellular transport of cargo and lipids from endoplasmic reticulum to the Golgi apparatus is mediated via vesicles generated by a set of cytoplasmic coat proteins known as the COPII coat. Building on a catalog of yeast mutants and in vitro reconstitution of ER–Golgi transport events, the COPII coat was initially defined more than 20 years ago (Fromme and Schekman 2005; Miller and Schekman 2013). Using cryo-electron microscopy and single-particle analysis, the structure of the Sec13/31 COPII coat cage was solved at 3 nm resolution (Stagg et al. 2006). Combining latest cryo-electron microscopy developments and mass spectrometry, a reliable pseudo-atomic model of the COPII cage was determined at 1.2 nm resolution, which could explain assembly and flexibility during coat formation (Noble et al. 2013). Using in vitro reconstitutions, the roles of COPII scaffold in remodeling the shape of a lipid bilayer were examined. The COPII proteins induced beads-on-a-string-like constricted tubules, similar to those previously observed in cells (Bacia et al. 2011). Unfortunately, the comparison of in vitro cryo-electron microscopy data with cellular electron microscopy was done with chemically fixed and resin-embedded samples. Cryo-fixed cells would have been favorable and desirable, as high-pressure freeze fixation combined with freeze substitution is nowadays a routine procedure in many laboratories. Of course, the closest approximation would have been cryo-electron microscopy or tomography of vitreous sections (CEMOVIS/CETOVIS), where such tubules formed like beads-on-a-string and emanating from endoplasmic reticulum have not been reported yet.

An impressive example of studying Golgi-related mechanisms by in vitro approaches is the assembly of the SNARE complex on membrane fusion. Cellular membrane fusion is thought to proceed through intermediates including docking of apposed lipid bilayers, merging of proximal leaflets to form a hemifusion diaphragm, and fusion pore opening. Recently, the SNARE fusion machinery was arrested in a cell-free reaction, and the fusion intermediates were identified by cryo-electron microscopy (Hernandez et al. 2012). Currently, such data derived from cellular cryo-electron microscopy are lacking. But it is rather a question of time than feasibility until research on frozen-hydrated cells will show such docking and fusion events at convenient resolution and in their cellular context.

In sub-tomogram averaging, features of cryo-electron tomography are combined with single-particle reconstruction procedures to provide 3D information and structural information of macromolecular complexes in situ. Multiple copies of certain macromolecular complexes are identified in cryo-electron tomograms. Then, these sub-tomograms containing the complex of interest are extracted from the larger original data set, aligned and averaged to obtain an isotropic 3D structure of the complex. One of the first examples was the reconstruction of nuclear pores in *Dictyostelium discoideum* (Beck et al. 2004). Recent applications provided resolutions of 2–4 nm on samples including polysomes, nuclear pore complexes, viral proteins, flagella, microtubule binding proteins, respiratory chain complexes, chromatin, chemoreceptor arrays, and desmosome plaques (reviewed in: Briggs 2013). Many of these applications involve membrane-bound complexes, which are particularly challenging to study by other structural biology methods. The majority of successful applications of sub-tomogram averaging focused on large complexes located inside cells or organelles (typically above 750 kDa), small complexes located on the surface of viruses or vesicles (typically above 300 kDa), or smaller complexes that assemble into regular arrays such as viral structural lattices. Regarding the secretory pathway, a very impressive recent study reconstructed individual COPI-coated membrane vesicles assembled in vitro (Faini et al. 2012). The coatomer was observed to adopt alternative conformations to change the number of other coatomers with which it interacts and to form vesicles with variable sizes and shapes, representing a fundamentally different basis for vesicle coat assembly (Faini et al. 2013).

All the high-resolution studies mentioned above using single-particle reconstruction, in vitro reconstitution, and/or sub-tomogram averaging clearly gave great insights into protein functions, arrangements of protein complexes, and protein–membrane interactions. Hence, the determined 3D shapes could be used as patterns for finding similar protein complex shapes in reconstructed 3D volumes of vitrified Golgi stacks in their cellular context. The basic idea of ‘visual proteomics’ to map molecular landscapes inside unperturbed cellular environments into a quantitative description of macromolecular interactions that underlie cellular functions (Nickell et al. 2006) did obviously not generate (yet) new substantial knowledge on the secretory pathway including Golgi apparatus. Probably, the simultaneous combination with novel (genetic) tagging and labeling techniques in cellular cryo-electron microscopy (Bouchet-Marquis and Hoenger 2011; Bouchet-Marquis et al. 2012) will shed further light on the molecular interactions orchestrating a functional Golgi apparatus.

## Conclusions and outlook

This review shows that describing the secretory pathway in its native state by cryo-electron microscopy has already started. Regarding that functional elements like macromolecular complexes interact at Golgi apparatus in distances far below light microscopic resolution, and that vesicles or cisternae of different compositions are homogenized during biochemical isolation or fractionation, it is obvious that high-resolution imaging technologies like cryo-electron microscopy are necessary to decipher Golgi functionality. Most likely, this will not be solved using cellular cryo-electron microscopy as stand-alone technique, but rather in conjunction with various other technologies such as structural biology, biochemistry, light microscopy, and mass spectrometry-based proteomics.

In quantitative proteomic analyses, more than 1,400 different proteins have been reported to be involved in the early secretory pathway (Gilchrist et al. 2006; Au et al. 2007) and more than 60 proteins in clathrin-coated vesicle formation (Borner et al. 2006; Bergeron et al. 2010; McPherson 2010). Not one of them could be unambiguously identified in vitrified sections by pattern recognition. This might be due to the ‘non-optimal conditions’, typical for applying cryo-electron tomography to relatively complex and less abundant biological structures. However, the idea of describing Golgi architecture at the molecular level by means of ‘visual proteomics’ at quasi-atomic resolution (Nickell et al. 2006) might be difficult to achieve at this stage. On the other hand, the high-resolution studies using single-particle reconstruction and sub-tomogram averaging explained already molecular interactions in purified samples, which have to be retraced in reconstructed data of Golgi complex in its cellular context.

Current and new labeling techniques are on their way to find applications in vitreous samples (Bouchet-Marquis et al. 2012). Improvements on high-pressure freeze fixation and sample preparation in terms of reproducibility and increased freezing quality in combination with the dynamics of in vivo light microscopy and probably in direct correlation to cryo-electron microscopy will help identifying the key players of Golgi function in the future. Accordingly, with the constant improvements in cryo-electron tomography, such as microscope tilting stage stability at liquid nitrogen or helium temperature, reduction in radiation damage with the use of sensitive CCD or CMOS cameras, data denoising, and the use of energy filters to improve the signal-to-noise ratio, it is very likely that 3D reconstructions of the complete secretory pathway in vitreous ice will emerge (Bouchet-Marquis et al. 2008). The 3D reconstruction of large volume areas using focused ion beam (FIB-SEM)—currently in resin-embedded samples and probably soon in the cryo-state—will broaden the knowledge of how the Golgi apparatus in its entity interacts with the membranes of endoplasmic reticulum and other intracellular organelles including cytoskeleton. The fate of disintegrated Golgi membranes during mitosis and how the organelle is rebuilt in daughter cells will also be a question that might be answered by large volume reconstructions, potentially correlated with light microscopic data of live cell dynamics.

Even since electron microscopy gained substantial understanding on the complex Golgi morphology in the last six decades, we still cannot delineate a completely unperturbed ultrastructure of a functioning Golgi apparatus inside a living cell. The combination of electron and cryo-electron microscopy with novel super-resolution light microscopic approaches of live cells might help to answer such fundamental questions, which seemed so straight forward from the beginning.
